# Beta sitosterol and Daucosterol (phytosterols identified in *Grewia tiliaefolia*) perturbs cell cycle and induces apoptotic cell death in A549 cells

**DOI:** 10.1038/s41598-017-03511-4

**Published:** 2017-06-13

**Authors:** Tamilselvam Rajavel, Ramar Mohankumar, Govindaraju Archunan, Kandasamy Ruckmani, Kasi Pandima Devi

**Affiliations:** 1 Department of Biotechnology, Science Campus, Alagappa University, Karaikudi, 630 003 Tamil Nadu India; 2 National Facility for Drug Development (NFDD) for Academia, Pharmaceutical and Allied Industries, Bharathidasan Institute of Technology, Anna University, Tiruchirappalli, 620024 Tamil Nadu India; 3 Centre for Pheromone Technology, Department of Animal Science, Bharathidasan, University, Tiruchirappalli, 620 024 Tamil Nadu India

## Abstract

Lung cancer is the leading cause of cancer related deaths both in developed and developing countries. Since majority of the existing therapeutic methods harms both normal and malignant cells, a viable alternative is to switch into safe and beneficial traditional medicinal plants. Hence the present study was framed to identify selective anti-lung cancer agents from the medicinal plant *Grewia tiliaefolia* (GT). Cell viability experiments showed that benzene extract of GT (BGT) leaf effectively inhibited the growth of A549 cells, while being non-toxic to normal human lung L132 and PBMC cells. Ames and comet assays demonstrated that BGT is of non-mutagenic and non-genotoxic nature in untransformed cells. The hematological and histopathological profiles of the *in vivo* acute and sub-acute toxicity studies demonstrated that BGT is safe and tolerable. Importantly, western blot analysis and Annexin V-FITC staining confirmed that BGT promotes mitochondrial dependent apoptotic cell death in A549 cells by arresting cell cycle at G2/M phase. Bio-assay guided fractionation revealed the presence of phytosteols (β-sitosterol and daucosterol) which significantly inhibited the growth of A549 cells both alone and in combination. This study warrants that these phytosterols in alone or in combination can be considered as safe and potential drug candidates for lung cancer treatment.

## Introduction

In the last decades, cancer research has enormously increased due to the rapid increase of cancer related death around the world. According to the IARC data, cancer affects nearly 14.1 billion people and causes 8.2 million death worldwide, which has been statistically increasing from the year of 2008^1^. As per the GLOBOCAN report 2012, lung cancer is the most predominant and aggressive type of cancer which affects nearly 1.8 million people (per annum) in the world population^1^. Based on its histology lung cancers are categorized into two types: non-small cell lung cancer (NSCLC-more common) and small cell lung cancer (SCLC-rare). The principle factors involved in 85% of the lung cancer related death include smoking and exposure to environmental pollutions^2^. Though FDA has approved many small molecules and monoclonal antibodies as drugs against various human cancers, still cancer remains as an incurable disease. The reason is that the existing therapeutic protocols and knowledge fail to overcome drug resistance, side effects and reoccurrence of cancer. Hence improving the current therapeutics is the major concern in today’s context. Current chemotherapeutic methods use synthetic cytotoxic molecules to kill and cause cell death in rapidly dividing cancer cells which could also affect normal cells. On the other hand, rapidly emerging drug resistance further limits the therapeutic application of chemotherapeutical drugs. Hence in the current scenario, potential therapeutic agents are needed which could target only the cancer cells without causing harmful effects to the normal human cells.

In this regards natural products offers large platform for the development of new drugs or small molecules against cancers, which are safe and devoid of toxicity. Several anticancer agents were identified from natural sources like curcumin, vinblastin, etoposide, teniposide, camptothecin, docetaxel, paclitaxel, sulforaphane and so on. These are plant derived anticancer drugs which stops the tumor growth through various mechanisms^3^. Moreover 90% of the world population relies on plant based products for their primary health care. India and other Asian countries have large number of traditional knowledge against a wide range of diseases including cancer, but most of them are not yet scientifically evaluated. Hence to provide scientific evidence, the present study has been designed to screen the Indian traditional medicinal plant *Grewia tiliaefolia* leaf against human lung cancer cells and to identify the anticancer agents present in it.

*Grewia tiliaefolia* (GT) is a subtropical, medium sized tree which belongs to the family of Malvaceae and commonly found in many eastern parts of India, China and Australia. Different parts of this plant have been used to treat several human illnesses like jaundice, throat pain, wound healing, urinary infection, dysentery and so on^4, 5^. For instance, the bark extract of the plant possess hepatoprotective effect against CCl_4_ induced toxicity in rats and the two isolated constituents D-erythro-2-hexenoic acid γ-lactone (EHGL) and Gulonic acid γ-lactone (GAGL) showed strong antioxidant activities against free radicals^6^. In addition, the bark of the plant contain high amount of lupeol and betulin, which are the pharmacologically active triterpenoids demonstrated to possess a wide range of medicinal properties including anticancer effects^7^. Regarding the safety, recently our group have demonstrated that the methanolic leaf extract of GT is safe and non-toxic, when analysed using both *in vitro* and *in vivo* experimental models^8^. Subsequently we also identified that the active principle constituent vitexin exhibits cholinesterase inhibitory, anti-amyloidogenic and neuroprotective effects against Aβ_25–35_ induced neurotoxicity in N2A cells^9^. However the leaf of the plant has not been scientifically explored against human cancer, so far. Hence in the present study we have aimed to study the anticancer potential of GT leaf extract against human non-small cell lung cancer (NSCLC) A549 cells, elucidate its mechanism and identify the active principle compounds.

## Results

### BGT induces cytotoxicity in A549 cells

**Figure 1:**
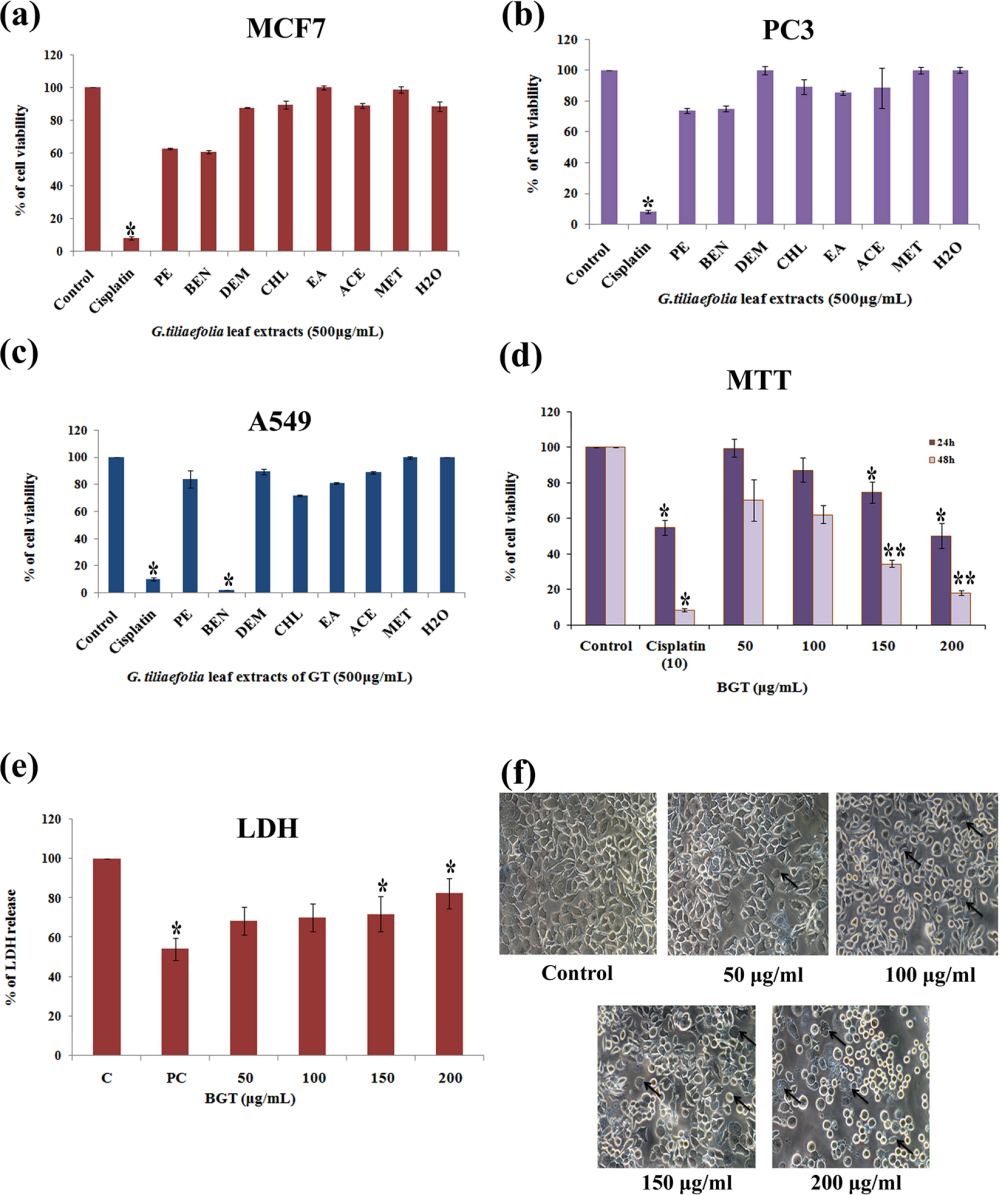
Cytotoxic effect of *Grewia tiliaefolia* (GT) leaf on A549 cells. (**a–c**) Cytotoxic effect of different solvent extracts of GT on MCF-7, PC-3 and A549 cells at 48 h by MTT assay. (**d**) Cytotoxic effect of various concentration of BGT on A549 cells at 24 h and 48 h. (**e**) LDH leakage in A549 cells during the treatment of BGT at indicated concentrations for 48 h. (**f**) Morphological changes of A549 cells during BGT treatment after 48 h. The arrow heads indicates that the cells undergoing apoptosis display a series of typical morphological features such as cell shrinkage, membrane blebbing, reduced cell intensity and formation of apoptotic bodies. The data are presented as the mean ± SD of three independent experiments. *P < 0.05, **P < 0.01 vs control group

Cytotoxic effect of various solvent leaf extracts of *G.tiliaefolia* on A549, PC3 and MCF-7 cells were evaluated by MTT assay and it  confirms that benzene extract of *G. tiliaefolia* leaf (BGT) significantly inhibits the proliferation of A549 cells by a concentration and time dependent manner when compared with the other solvent extracts. However, GT extracts does not significantly affect the growth of other cancer cell lines such as PC3 and MCF-7 (Fig. 1a and b). Indeed, most of the non-polar solvent extracts also exhibited cytotoxicity against A549 cells, however only at the maximum concentration [500 μg/ml] (Fig. 1c). Hence further anticancer studies were carried out with BGT fraction alone especially against A549 cells. The IC_50_ values of BGT on A549 cells were found to be 192.57 ± 5.22 and 121.12 ± 3.44 μg/ml at 24 and 48 h respectively (Fig. 1d). LDH assay further revealed that BGT significantly increased the LDH release dose dependently in A549 cells at 48 h. Consistent with the above results, cell viability was also affected from 50 μg/ml concentrations onwards. Importantly, 80% cell death was observed at 200 μg/ml by correlating with total LDH level in Triton-x treated cells (Fig. 1e). Taken together, MTT and LDH experiments suggested that the active ingredients presents in BGT has a strong cytotoxic effect on NSCLC A549 cells.

Cell morphology of BGT treated A549 cells exhibited cell shrinkage, irregular shape of epithelial morphology, rounded shape, reduced cell density and cell membrane blebbing after 48 h of exposure (Fig. 1f). Morphological changes positively indicated that BGT might promote apoptotic cell death in A549 cells.

### BGT is not harmful to normal human lung and PBMC cells

**Figure 2:**
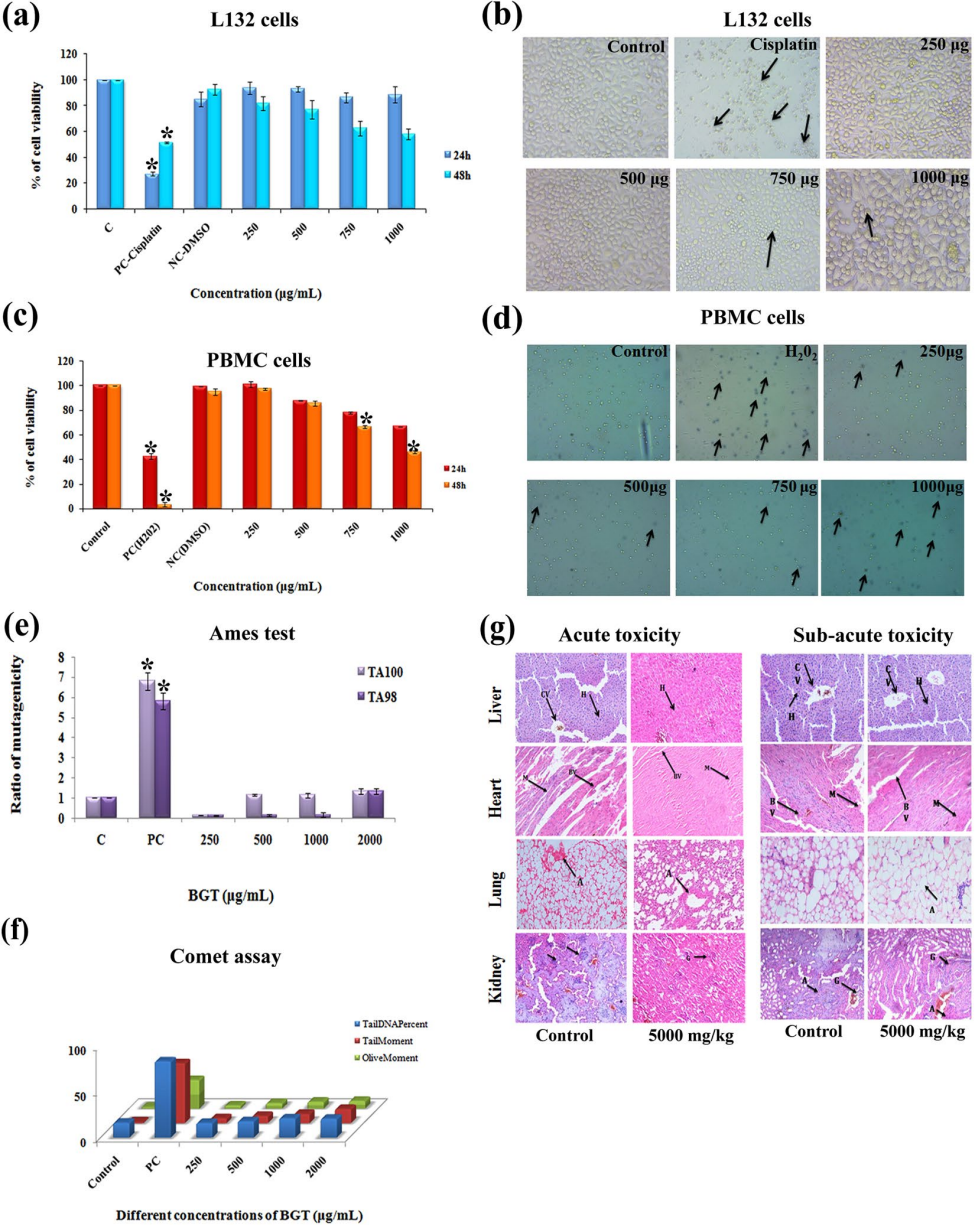
Pre-clinical safety effect of BGT in normal human cells and mice. (**a**) Cytotoxic effect of BGT on non-cancerous human lung L132 cells at 24 h and 48 h treatment. (**b**) Light microscopic images of control and BGT treated L132 cells exhibited healthy and normal epithelial morphology. (**c**) Cytotoxic effect of BGT on human PBMC cells at the indicated concentration for 24 h and 48 h. Cell viability was assessed by trypan blue dye exclusion method. (**d**) Light microscopic images of control and BGT treated PBMC cells and arrow heads indicates dead or dye included cells (**e**,**f**) Mutagenic and genotoxic effect of BGT on human PBMC cells after 48 h treatment. Mutagenicity and genotoxicity was assessed by ames and comet assays respectively. (**g**) Histopathological images of BGT treated mice tissue samples by acute and sub-acute toxicity studies. CV-Central Vein, H-Hepatocytes, BV-Blood Vessels, M-Myocardium, A-Alveoli, C- Cortex, G – Glomerulus, A- Artery (Hematoxylin-Eosin stain, 200X). *p < 0.01 denote the statistical significance between the control and treated groups.

Cytotoxic effect of BGT on normal human lung (L132 Cells) and PBMC (Peripheral Blood Mononuclear Cell) cells were examined by MTT and trypan blue dye exclusion method respectively. The results revealed that BGT did not affect the growth and viability of L132 cells at 24 h. However when the treatment time was increased to 48 h, it caused mild toxicity (40%) on L132 cells (Fig. 2a). Interestingly, the cytotoxic concentration of BGT in normal lung cells was 4X times higher than the effective concentration for A549 cells. However, few L132 cells exhibited shrinkage and change in cell morphology when treated at the maximum concentration of BGT [1 mg/ml] (Fig. 2b).

Similar to the effect observed in normal lung cells, cytotoxic effect of BGT in human PBMC cells were also observed only when the cells were treated with the maximum concentration of BGT (1 mg/ml) and the IC_50_ was observed to be 951.39 ± 8.3 μg/ml at 48 h (Fig. 2c and d). The cytotoxic concentration of BGT in human PBMC cells was actually different from the effect observed in both A549 and L132 cells. Overall, the cytotoxic experiments on normal human cells revealed that BGT exhibit selective cytotoxicity on A549 cells alone, sparing the normal cells.

### Genotoxicity and mutagenicity of BGT

Ames test was performed to assess the mutagenic potential of BGT on two histidine auxotrophic bacterial strains TA98 and TA100. The results (Fig. 2e) indicate that BGT at the tested concentration (250–2000 μg/ml) does not show any mutagenic effect in the tested bacterial strains TA98 and TA100 (RM < 2), when compared to that of the positive controls Sodium azide and 4-Nitroquinoline. The results again substantiate the safety of BGT and its constituents due to its non-mutagenic nature. In addition, Comet assay was performed in untransformed PBMC cells to detect the DNA damage caused by BGT (Fig. 2f). BGT (250–2000 μg/ml) did not induce any significant DNA damage in PBMC cells when compared with the H_2_O_2_ treated group. Together, the data confirmed that BGT and its constituents were of non-mutagenic and non-genotoxic nature in normal human cells.

### *In vivo* safety effect of BGT

Evaluation of pre-clinical safety of any drug is highly significant in the drug development process before it is tested for human uses. Hence the safety of BGT was performed in Swiss albino mice through acute and sub-acute toxicity testing methods. The results of acute toxicity testing of BGT observed that the animals were healthy without showing any significant changes in the behavior and mortality rate. In addition, hematological profile of BGT treated mice was also found to be in the normal range when compared with the control animals (Table 1). Importantly, no significant changes were observed in liver and kidney parameters like SGOT, SGPT, ALP, creatinine and urea. Similarly the lipid profile of BGT treated groups were also observed within normal ranges. Further, histopathological examination of lungs, heart, liver and kidney showed almost normal histology even at the maximum dose of BGT [5 g/kg body weight] (Fig. 2g). In case of sub-acute toxicity study, the animals in the BGT treated groups were found to be healthy and no significant changes were observed in both hematological and liver parameters (Table 1). However analysis of lipid profile showed that there was a significant reduction in total cholesterol level, which might be due to the cholesterol lowering effect of the active compounds present in BGT. Histopathological examination of sub-acute toxicity study also revealed that the tissues showed normal histo-architecture in BGT treated groups (Fig. 2g). Overall, *in vivo* toxicity experiments indicated that the active compounds present in BGT are safe, non-toxic in nature and has cholesterol lowering effect.

**Table. 1:** Hematological and biochemical profile of acute and sub-acute toxicity studies on swiss albino mice treated with BGT.

Biochemical and haematological Parameters	Acute Toxicity		Sub-acute toxicity	
	Control	5000 mg/kg of body weight	Control	5000 mg/kg of body weight
Sugar (mg/dL)	101.25 ± 2.59	103.25 ± 3.01	110.5 ± 2.33	113.2 ± 5.79
HB (gm %)	14.20 ± 1.20	13.13 ± 0.52	14.10 ± 0.72	13.63 ± 1.04
WBC (10^3^/mm^3^)	6.8 ± 0.14	7.2 ± 0.43	8.3 ± 481.32	8.5 ± 355.90
RBC (106/mm3)	4.75 ± 0.41	4.48 ± 0.27	4.68 ± 0.23	4.53 ± 0.33
SGOT (U/L)	67.50 ± 6.81	67.0 ± 1.83	41.75 ± 4.27	62.25 ± 17.31
SGPT (U/L)	44.50 ± 2.33	43.00 ± 3.54	24.75 ± 6.34	34.00 ± 18.76
ALP (U/L)	99.25 ± 5.28	102.75 ± 1.44	98.25 ± 9.29	119.75 ± 24.50
Urea (mg %)	37.25 ± 1.55	38.50 ± 1.04	32.38 ± 10.58	65.40 ± 3.69
Creatinine (mg %)	0.28 ± 0.09	0.4 ± 0.09	0.30 ± 0.04	0.45 ± 0.10
Protein (g %)	13.08 ± 0.65	13.63 ± 1.04	4.05 ± 0.33	4.93 ± 0.18
Albumin	7.68 ± 0.40	8.10 ± 0.51	2.73 ± 0.28	3.33 ± 0.20
Cholesterol (mg/dL)	135.25 ± 11.6	135.25 ± 5.57	47.00 ± 3.56	22.25 ± 19.95
TGL (mg/dL)	73.25 ± 5.63	77.00 ± 5.21	39.50 ± 1.26	42.00 ± 4.02
HDL (mg/dL)	21.50 ± 2.10	19.75 ± 1.11	9.00 ± 1.08	7.50 ± 0.96
LDL (mg/dL)	86.70 ± 7.65	84.18 ± 6.53	28.25 ± 4.08	43.30 ± 7.52
VLDL (mg/dL)	27.05 ± 2.33	27.05 ± 1.11	7.75 ± 0.34	8.40 ± 0.80

The results are expressed as mean ± SD.

### BGT causes DNA fragmentation in A549 cells

**Figure 3:**
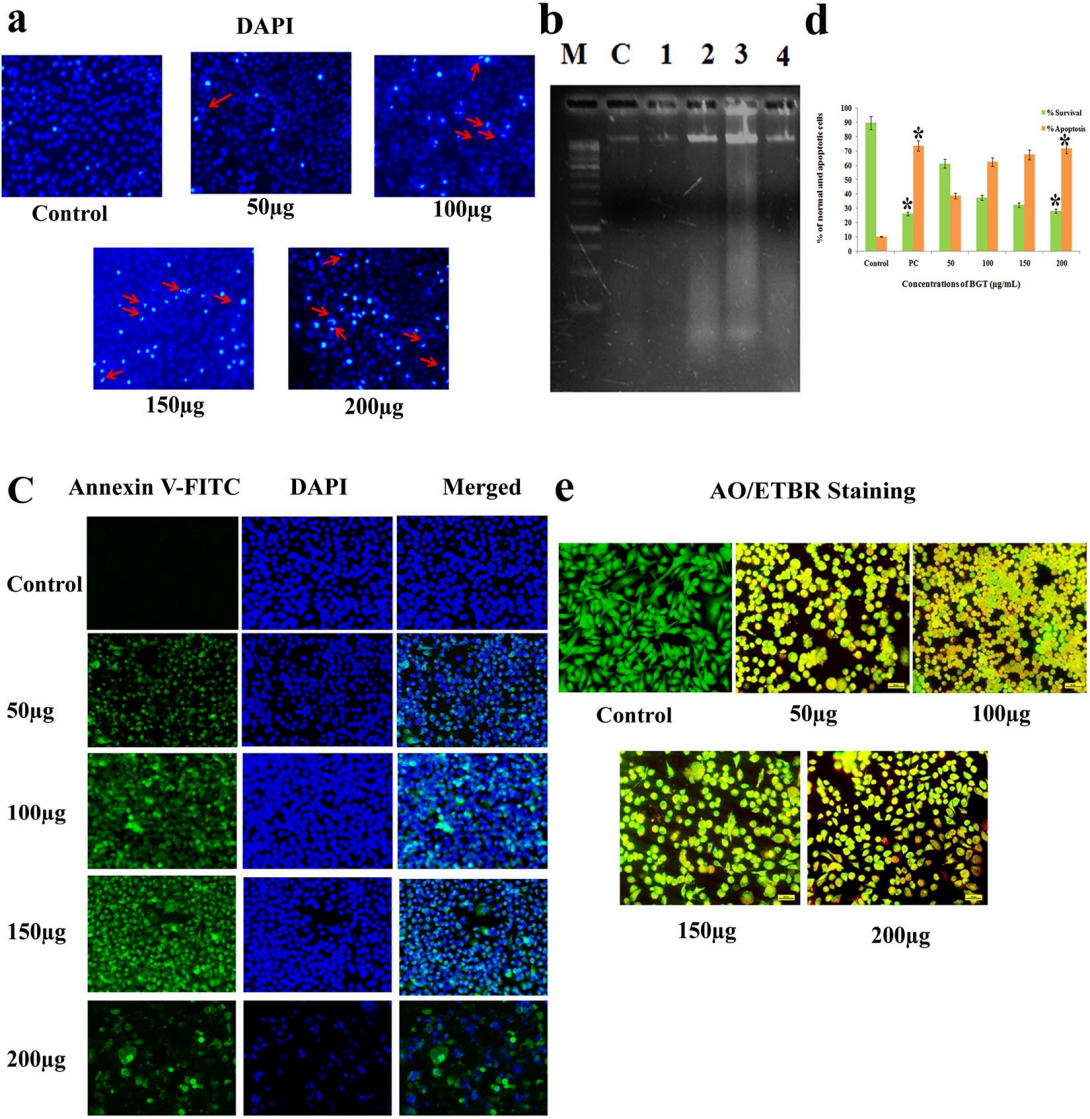
BGT induced apoptosis in A549 cells. (**a**) A549 cells treated with indicated concentration of BGT for 48 h, stained by DAPI and observed in fluorescence microscopy. The arrow heads indicates chromatin condensation with changes of nuclear morphology and DNA fragmentations. (**b**) DNA fragmentation of BGT treated A549 cells. M-1kb ladder DNA, C-Control and Lane 1–4 is different concentration of BGT (50–200 μg/mL) treated A549 cells. (**c**) Fluorescence microscopy images of Annexin-V/FITC and DAPI stained BGT treated and control cells. (**d**) Quantitative measurement of apoptotic cells after BGT treatment with the indicated concentration at 48 h. (**e**) Fluorescence microscopy images of AO/ETBR stained BGT treated A549 cells after 48 h. All the images were acquired in 200X magnification. *p < 0.01 denote the statistical significance between the control and treated groups.

To test whether the decrease in cell viability was due to the apoptotic effect of BGT, A549 cells were stained with DAPI stain. Fluorescence microscopic images of BGT treated A549 cells showed nuclear fragmentation, irregular nuclear morphology, nuclear membrane disruption, condensed chromatin and intense fluorescence in the nucleus of A549 cells (Fig. 3a). In addition, the agarose gel picture showed (Fig. 3b) ladder pattern of DNA in BGT treated A549 cells. However, these apoptotic features were not observed in the control cells. These changes are generally associated with cells undergoing apoptotic mode of cell death^10, 11^. The results of DNA fragmentation and DAPI staining revealed that BGT promotes apoptotic mode of cell death in A549 cells.

### BGT induced apoptotic cell death in A549 cells

The apoptotic effect of BGT was confirmed by Annexin V-FITC/DAPI double staining using fluorescence microscopy analysis. Translocation of phosphatidylserine from inner to outer cell membrane is a significant marker in early apoptotic cells. Fluorescence microscopy analysis revealed the presence of strong fluorescence on the surface of (Fig. 3c) BGT treated A549 cells and the number of fluorescence positive cells increased in a dose dependent manner. However, complete absence of Annexin V-FITC staining was observed in control cells. The results demonstrated that BGT promotes apoptotic mode of cell death in A549 cells. The result was also substantiated with AO/EtBr dual staining method. Both, fluorescence spectrophotometer results and fluorescence images revealed that BGT promotes apoptotic cell death in A549 cells by a dose dependent manner (Fig. 3d and e). Together, the results suggested that BGT induced cell death in A549 cells via apoptosis rather than necrotic mode.

**Figure 4:**
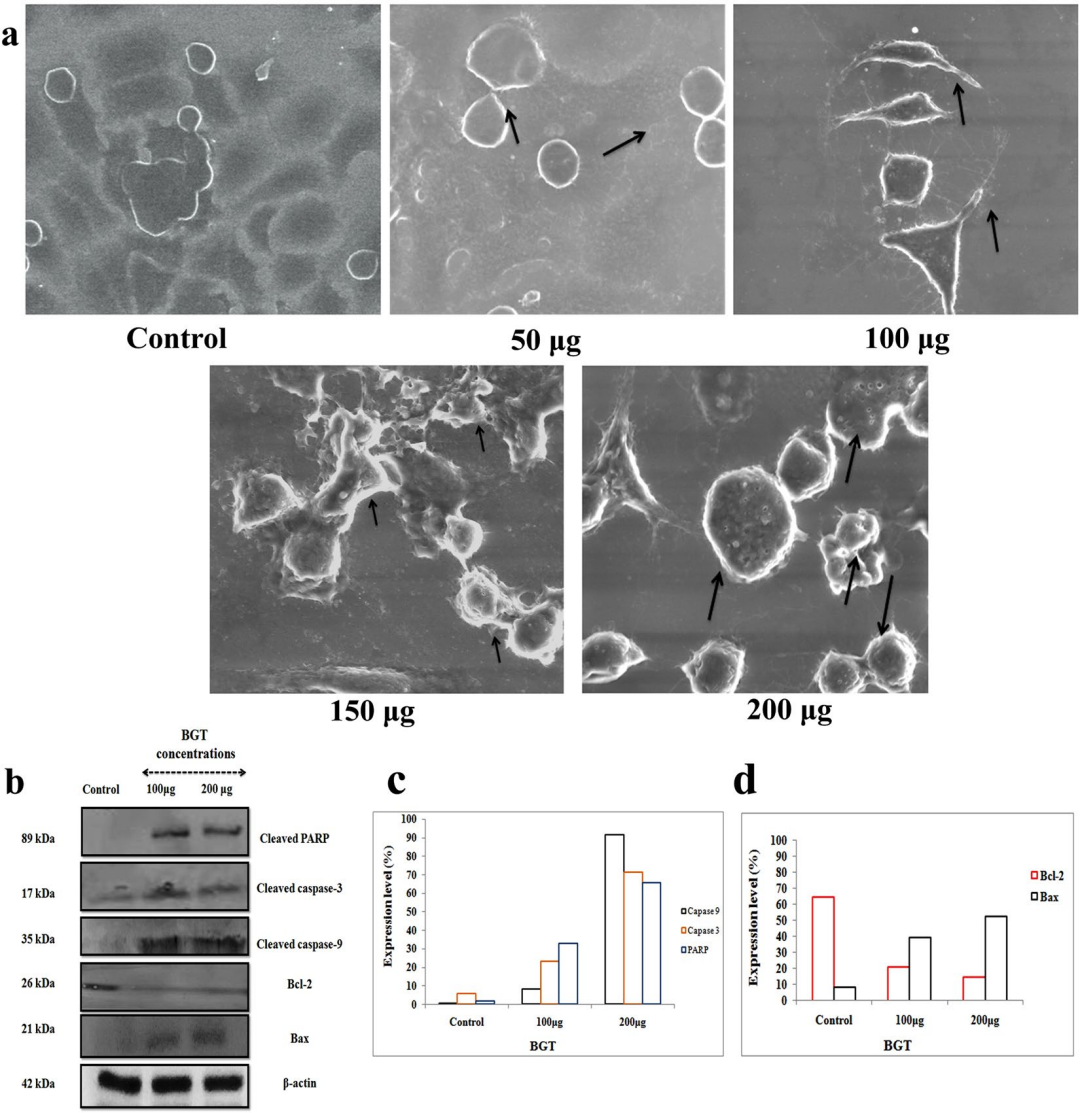
BGT promotes caspase dependent apoptosis in A549 cells. (**a**) Scanning electron microscopic images of A549 cells treated with indicated concentration of BGT at 48 h. (**b**) western blot analysis of BGT treated A549 cells indicated concentration at 48 h incubation. β-actin was used as internal control and expression level of cleaved PARP, cleaved caspase-3, cleaved caspase-9, Bcl-2 and Bax was studied in BGT treated and untreated groups. (**c**) Expression level of intrinsic apoptotic proteins that was measured using ImageJ software analysis. (**d**) Expression level of pro and anti-apoptotic proteins.

The cells which undergo apoptosis usually displays distinct morphological changes like apoptotic bodies, altered microvilli and membrane blebbing that were demonstrated by Scanning Electron Microscopy. SEM analysis showed (Fig. 4a) severely altered microvilli, formation of apoptotic bodies, complete absence of microvilli and cell membrane blebbing in BGT treated groups. However the control cells exhibited typical epithelial shape and high population of cells.

### BGT activates intrinsic apoptotic pathway

To explore the molecular mechanism by which BGT induced apoptosis, the expression level of apoptotic proteins such as Bcl-2, Bax, PARP cleavage, Caspase-3 cleavage and caspase-9 cleavage were assessed through western blot analysis. The results showed that BGT treatment caused decreased expression of Bcl-2 (Fig. 4b and c) and upregulated the expression of Bax protein. It resulted in activation of caspase-3 cleavage which shows that the growth inhibitory effect of BGT was primarily due to apoptosis. The results were also substantiated by cleavage of PARP protein (Fig. 4b and d). Cleaved form of PARP protein was found in BGT treated A549 cells, which confirmed that BGT induces caspase dependent apoptosis in A549 cells. The expression of caspase-9 was studied to assess the mitochondrial apoptotic pathway in the cells. The cleaved form of caspase-9 was found in BGT treated groups, however it was absent in the control cells (Fig. 4b and d). Overall, western blot analysis confirmed that BGT promotes intrinsic mode of apoptotic cell death in human NSCLC A549 cells.

### BGT induced mitochondrial membrane depolarization and releases cytochrome c

**Figure 5:**
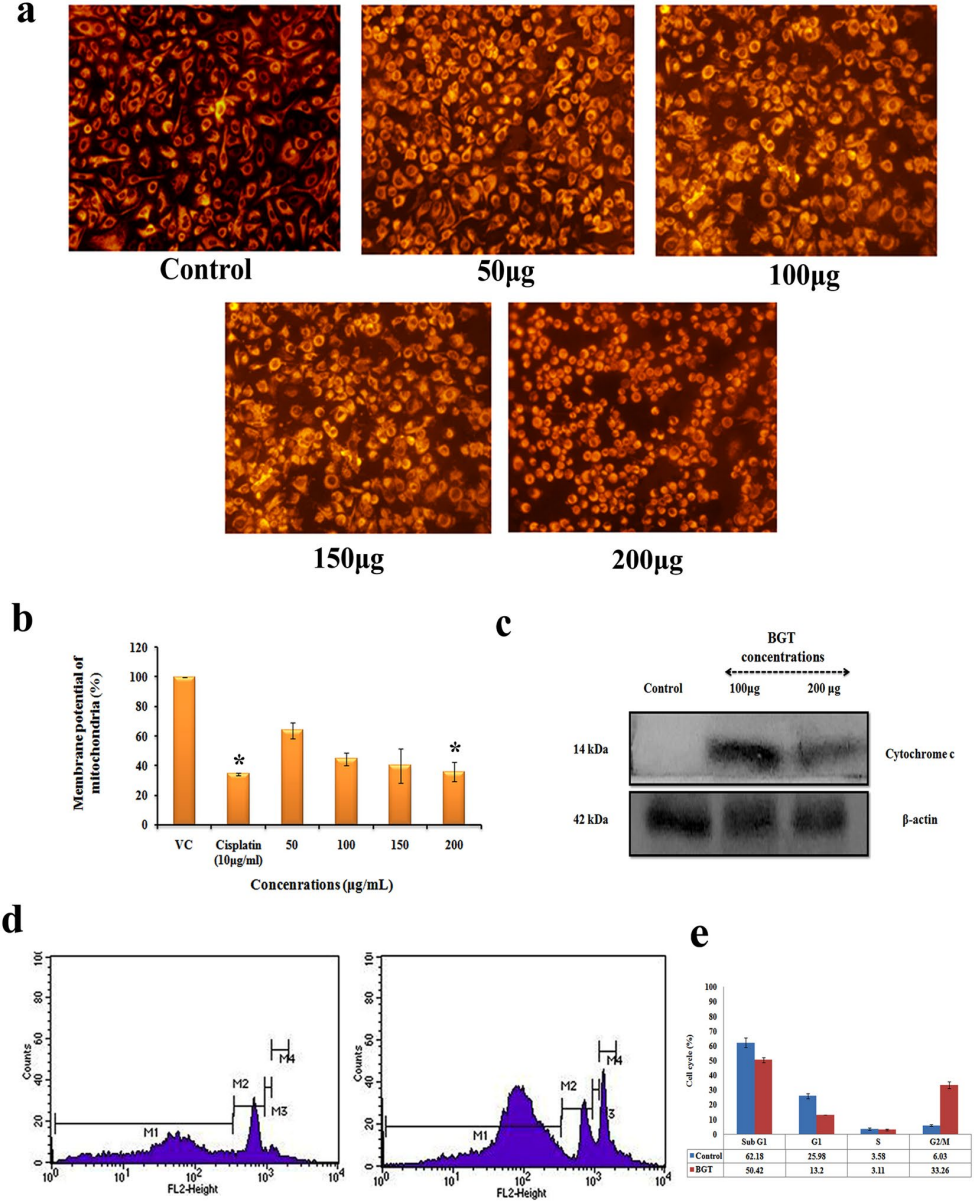
BGT causes mitochondrial membrane depolarization on A549 cells and causes G2/M phase arrest. (**a**) Rhodamine 123 stained A549 cells were treated with indicated concentration of BGT for 48 h and analysed in fluorescence microscopy. (**b**) Bar diagram shows the percentage of cells with disrupted MMP after BGT treatment. (**c**) Western blot analysis of BGT treated A549 cells at indicated concentration for 48 h. (**d**) BGT induced G2/M phase arrest in A549 cells. (**e**) Histogram shows the percentage of cells in G0/G1, G2/M, and S phase, after treatment with BGT.

Disruption of active mitochondria in cancer cells leads to intrinsic mode of apoptotic cell death^12^ which was assessed by Rhodamine 123 staining. The results of fluorescence spectroscopy analysis of the stained cells showed (Fig. 5a) a dose dependent reduction in the fluorescent intensity in BGT treated cells. Further, fluorescence images revealed loss of MMP in BGT treated group (Fig. 5b). Additionally, western blot analysis showed an upregulation of cytochrome c release (Fig. 5c) in BGT treated group, which was absent in the control cells. Together, the results suggest that BGT promotes apoptotic cell death in A549 cells by mitochondrial dependent pathway.

### BGT causes cell cycle arrest at G2/M phase

Since BGT exhibited selective cytotoxicity against lung cancer A549 by promoting apoptotic mode of cell death, we further analyzed the cell cycle distribution in the presence of BGT. Interestingly, after 48 h treatment with BGT (200 μg/ml) a dramatic accumulation of cells in subG1 phase were found, which is a known hallmark for the cells undergoing apoptosis (Fig. 5d). In addition, accumulation of cells in G2/M phase also increased upon BGT treatment (Fig. 5e), which suggested that cells may undergo mitotic arrest. Together, the results suggest that cell growth inhibition in BGT treated A549 cells might be due to mitotic arrest followed by apoptosis induction.

### β-Sitosterol and Daucosterol: active principles from BGT

**Figure 6:**
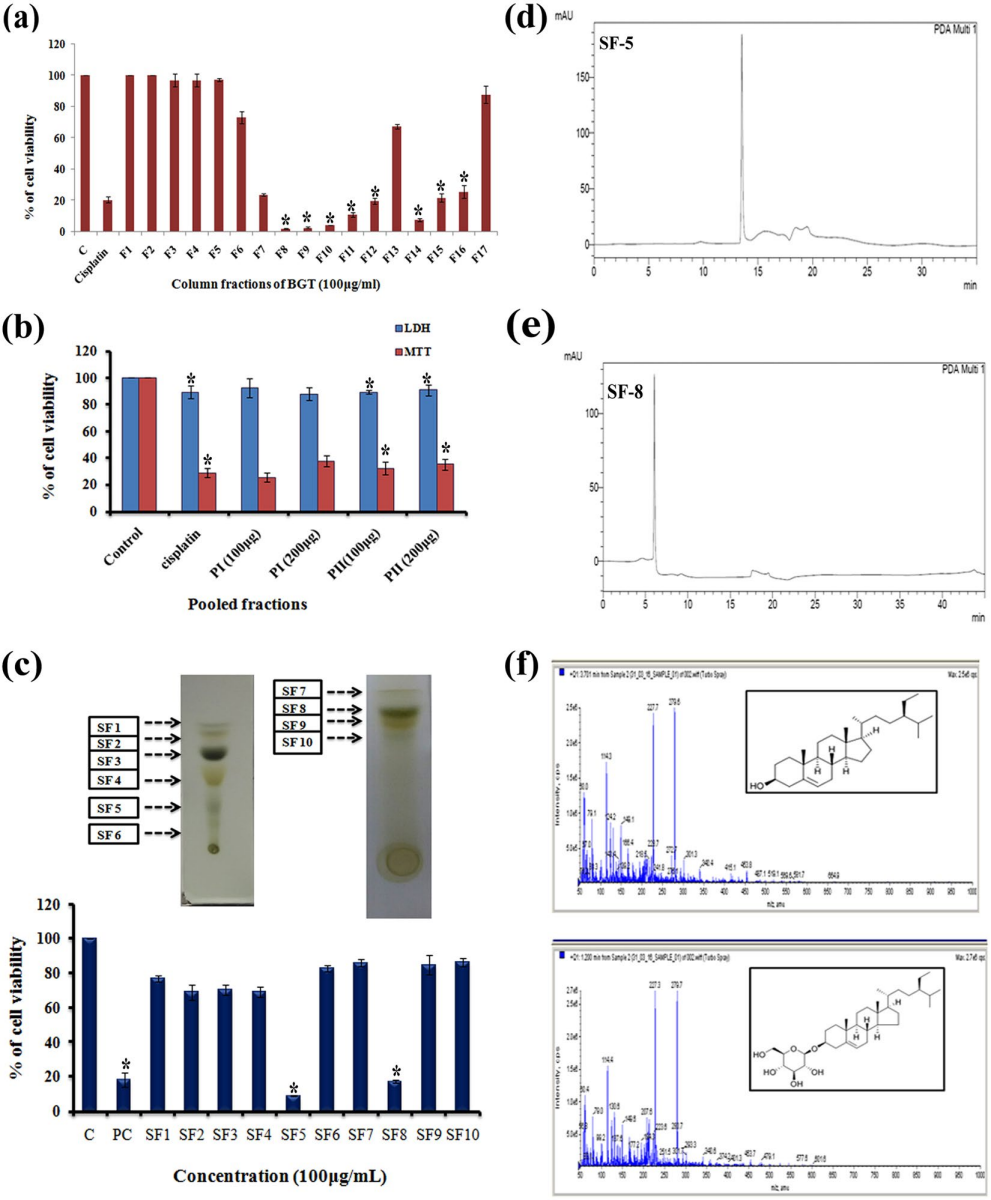
Identification of active principle compounds by bio-activity guided fractionation approach. (**a**) Fractionation of BGT by silica column chromatography with solvents of increasing polarity and cytotoxic evaluation of the column fractions in A549 cells by MTT assay. (**b**) Cytotoxic effect of active pooled fractions P1, P2 on A549 cells after 48 h treatment with indicated concentrations. (**c**) Sub-fractionation of BGT by thin layer chromatography. Cytotoxic effect of BGT sub-fractions on A549 cells after 48 h treatment. (**d**,**e**) Purity assessment of active BGT extracts SF-5 and SF-8 by HPLC analysis. (**f**) LC-MS-MS analysis of SF-5 and SF-8. *p < 0.01 denote the statistical significance between the control and treated groups.

Bio-activity guided fractionation was performed to identify the active principle compounds from BGT. After fractionation, all the column fractions (totally 17 fractions) were tested against A549 cells by MTT assay. The result shown in Fig. 6a indicates that fractions F-8, F-9, F-10, F-14 (100 μg/ml) exhibited significant cytotoxic effect against A549 cells. Hence these fractions were pooled as P1 (F-8 & 9), P2 (F-14 & 15) and further evaluated by MTT and LDH assay. A strong growth inhibition (Fig. 6b) was exhibited by both P1 & P2 (10 μg/ml). Moreover, similar IC_50_ values were found for both the pooled fractions. The pooled fractions were further sub-fractionated with TLC and 10 sub-fractions (SF) were obtained (Fig. 6c). Among which, SF-5 & 8 showed significant activity and it was subjected to HPLC analysis followed by LC-MS-MS analysis (Fig. 6d–f).

**Figure 7:**
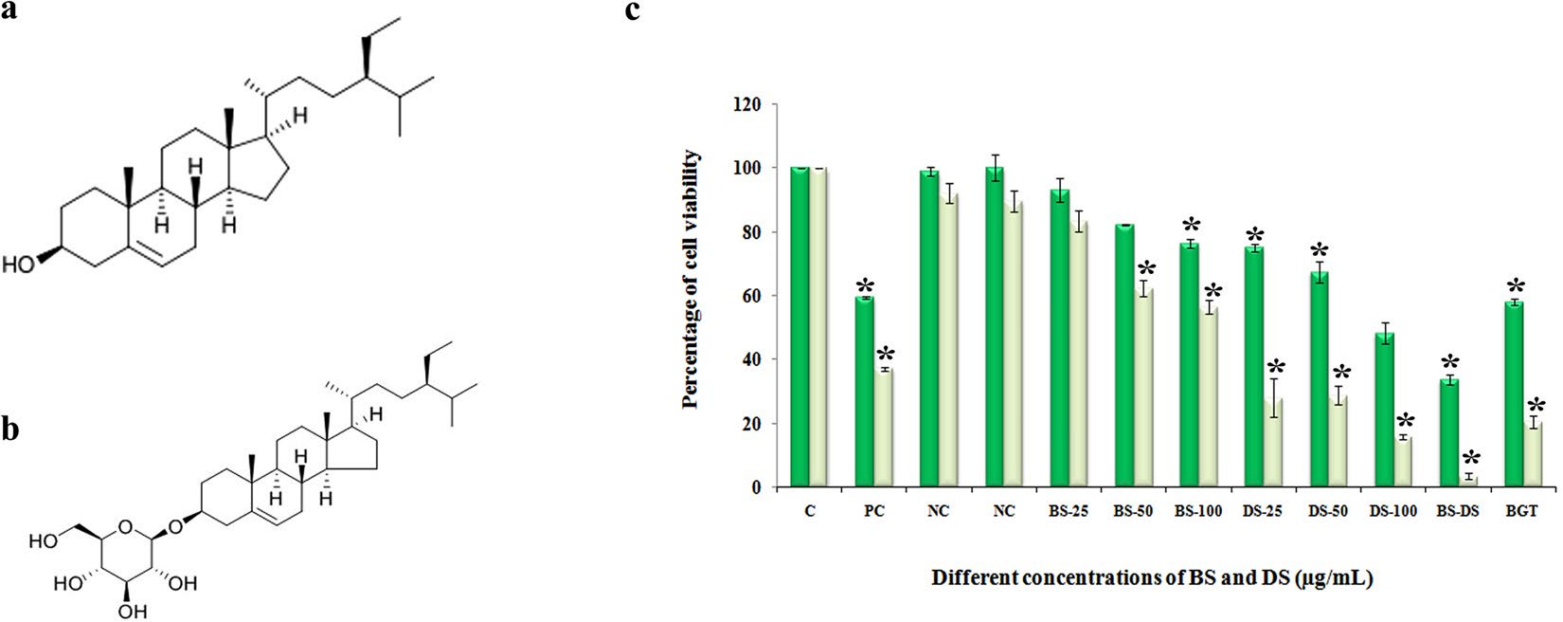
Beta–sitosterol and daucosterol are active principles BGT. (**a**) Chemical structure of beta sitosterol - PubChem CID: 222284 (**b**) Chemical structure of daucosterol – PubChem CID:5742590 (**c**). Cytotoxic effect of beta-sitosterol, daucosterol and its combination (1:1) against A549 cells after 24 and 48 h by MTT experiment.

Phytochemical profile of P1 revealed the presence of three compounds namely β-sitosterol, Daucosterol and D-gluconic acid. Moreover, same compounds were also identified in P2 except D-gluconic acid. The chemical structures of both compounds were depicted in Fig. 7a. The anticancer effects of identified compounds (β-Sitosterol and Daucosterol) were assessed by MTT assay and compared with the standard drug cisplatin. Both β-sitosterol (BS) and daucosterol (DS) significantly inhibited the proliferation of A549 cells by a dose and time dependent manner. However, IC_50_ concentration of DS was found to be 95.19 and 17.46 μg/ml at 24 and 48 h respectively (Fig. 7c). Daucosterol exhibited higher anti-cancer property than β-sitosterol and cisplatin. In addition, combination of β-sitosterol and daucosterol (1:1) showed increased anticancer activity in A549 cells.

## Discussion

Drugs from the plant sources have contributed towards remarkable application in cancer research for the last twenty years^13^. A large number of phytochemicals have been demonstrated to exhibit anticancer activities by activating apoptosis in cancer cells^14^. Promoting apoptosis in cancer cells is considered as an attractive therapeutic strategy in anticancer chemotherapy. Apart from their pro-apoptotic effect, studies on their molecular mechanisms have suggested that these phytochemicals targets many therapeutically important signaling pathways in cancer cells^15^. Consequently, plant based drug discovery has attracted many scientific communities in the last decades for developing drugs against human cancers. In the present study we have attempted to study the anticancer property of *G.tiliaefolia* leaf, an indigenous medicine which possesses various pharmacological activities. Through the cytotoxic experiments it was observed that the benzene extract of the plant (BGT) has significant anti-proliferative effect against A549 cells without causing any harmful effect to the normal human lung (L132) and PBMC cells. Cytotoxic concentration of BGT on L-132 and PBMC cells significantly varied from the effective concentration exhibited against the lung cancer A549 cells. The observed differential cytotoxic effect suggest that the active constituents present in BGT has selective cytotoxicity towards lung cancer cells alone. On the other hand, it was observed that cisplatin significantly induced cell death in both A549 and L132 cells. Compared to cisplatin, BGT may be a preferred drug on the basis of its lesser cytotoxic effect exhibited in normal lung cells. Further, the release of LDH to the cell supernatant in BGT treated A549 cells directly correlates with the loss of cell viability and cell membrane damage^16^. Results of both MTT and LDH assays confirmed that BGT significantly affects the proliferation of A549 cells and alters the cell membrane integrity. This is considered as a significant effect for any anti-cancer drug since loss of cell membrane integrity correlates with the late event in cells undergoing apoptosis as well as necrosis^17^.

The current cancer chemotherapy is very expensive and most of them are not safe for cancer patients. Preventing the side effects may reduce the clinical burdens and will have considerable economic implications^18^. In this regard, the safety effect of BGT was assessed through *in vitro* and *in vivo* experiments. Initial cytotoxic experiments showed that BGT exhibited less cytotoxic effect in normal human lung and PBMC cells. It confirmed that BGT possess selective cytotoxicity towards NSCLC A549 cells. Many natural products such as curcumin, resveratrol, genistein have shown less cytotoxic effect in normal cells and higher cytotoxic effect in cancer cells^19^. The results reveal that the constituents present in BGT were non-toxic to the normal human cells at the tested concentration. Since evaluation of genotoxicity and mutagenicity is one of the most important concerns for commercialization of drugs^20^, comet assay was performed, which revealed that no genotoxic or DNA damaging effect was induced by BGT in normal human PBMC cells. Moreover, ames test also revealed that BGT does not have any mutagenic effect in the tested bacterial strains. Overall, the *in vitro* experiments disclosed that BGT is safer and its constituents could be used as potential source for the development new anticancer drug which will be devoid of toxicity. The US Food and Drug Administration (FDA) emphasize the necessity of animal toxicity profile for screening new pharmacological drugs. Hence, *in vivo* safety of BGT was performed by acute and sub-acute toxicity testing in swiss albino mice. BGT treatment did not produce any mortality or behavior changes in both acute and sub-acute toxicity experiments. Moreover, no significant changes were observed in both blood and hematological parameters. In addition, histopathological analysis also showed normal tissue morphology without any necrotic or neoplastic signs. Hence, through both acute and sub-acute toxicity experiments we found that BGT and its constituents are safe for human use and can be considered as a potential source for development of anticancer drugs.

Apoptosis is a crucial phenomenon in the cytotoxic mechanism of many anticancer drugs. In the current study, a change in the cell morphology was observed in BGT treated A549 cells including cell shrinkage, elongation, reduced cell density and appearance of many mitotic cells (spherical shape)^21^. The morphological changes positively indicated that A549 cells undergo apoptotic mode of cell death during BGT treatment, which may be mediated through mitotic arrest. The apoptotic cells undergo DNA fragmentation and chromatin condensation due to the caspase activated DNase^22^. DAPI staining further confirmed that the apoptotic effect of BGT is accompanied by the presence of nuclear condensation, irregular morphology of nucleus and fragmentation of nuclei. DNA fragmentation was also demonstrated in agarose gel electrophoresis. Ladder pattern of low molecular weight DNA (Oligonucleosomal size fragments) is the unique biochemical feature in late apoptotic cells^23, 24^. BGT treated cells exhibited ladder pattern of DNA in low molecular weight that may be due to caspase activated DNAse^22^. Together, both DAPI and DNA fragmentation assay indicated that BGT cause apoptotic mode of cell death in A549 cells. The translocation of phosphatidylserine serine from inner membrane to outer membrane surface is a marker of apoptotic cell death^25^, which was assessed through Annexin V-FITC staining. Fluorescence microscopic images showed that BGT treatment caused strong fluorescence in the cell surface of A549 cells which indicated the accumulation of phosphotidylserine. The results suggested that growth inhibition of BGT against A549 cells was due to apoptosis. In addition, the results were also substantiated by AO/ETBR dual staining that indicated BGT promotes both early and late apoptosis dose dependently, without causing necrotic cell death in A549 cells. Overall, the mode of BGT induced cell death in A549 cells was confirmed to be apoptosis rather than the other kind of cell death.

Caspases are endolytic protease enzymes which play a crucial role in execution of mammalian cell death programmes like apoptosis^26^. Among them, caspases-3 is the commonly activated protease which catalyses the many key cellular proteins^27^. However, activation of pro-caspase 3 into executioner caspase 3 requires specific cleavage for its activation. Hence the expression of cleaved caspase-3 was studied in BGT treated A549 cells by western blot analysis. Interestingly, BGT treated groups exhibited cleaved caspase-3 (17 kDa) which was absent in the control cells. It confirmed that BGT induces caspase dependent apoptotic cell death in A549 cells. Additionally we also studied cleavage of PARP protein during BGT treatment which is one of the known substrate for caspases^28^. Similar to caspase-3, PARP was also cleaved (86 kDa) in the BGT treated groups which also confirms activation of caspase-3. Apoptotic programmed cell death is further classified into extrinsic and intrinsic or mitochondrial apoptotic pathway. However, both pathways have been executed by different effective caspases like caspase-8 and 9 respectively^29^. In this regard, we studied the effect of BGT on caspase-9 activation by western blot analysis. The result clearly confirms BGT activates mitochondrial dependent apoptosis in A549 cells by promoting the expression of caspase-9. However, mitochondrial membrane potential (MMP) is a crucial factor for the intrinsic apoptotic pathway and disruption of MMP leads to release of their components in the cell cytoplasm. Hence, we initially studied the MMP potential by Rhodamine 123 staining. Both fluorescence image and spectrophotometer analysis confirmed a reduction in the MMP during BGT treatment on A594 cells. It positively indicated that the apoptotic effect of BGT is mediated by mitochondrial dependent pathway. Hence, we further focused on elucidation of the role of BGT in regulating the mitochondrial mediated pathway through analyzing the expression level of Bcl-2, Bax and cytochrome c. Western blot results showed that BGT activates the release of cytochrome c, which upregulates the Bax protein (Pro-apoptotic) expression and decreased the expression of Bcl-2 protein (Anti-apoptotic). Cytochrome c is the initiator of intrinsic mediated apoptotic pathway which is normally present in mitochondrial membrane space and released into cytosol during apoptosis^30^. Western blot results substantiates that BGT promotes the release of cytochrome c into cytosol thereby mediating its apoptotic effect in A549 cells. In addition oncogenic expression of Bcl-2 protein plays crucial role in survival mechanism of cancer cells. Moreover, it is one of the challenging targets in anticancer drug discovery^31^. In this regard, the decreased expression of Bcl-2 and upregulation of Bax confirms that BGT also affects the survival mechanism of lung cancer A549 cells. Overall, the results revealed that BGT induces apoptosis via mitochondrial dysregulation in human NSCLC A549 cells.

Cell cycle is an essential process for the growth, development and differentiation of mammalian cells^32^. This process is strictly controlled by large number of proteins which are frequently dysregulated during malignant conditions^33^. In addition, many plant derived drugs block cell cycle progression at various stages of cell cycle such as G0/G1, S or G2/M phases and then induce apoptotic cell death^34^. Our data suggest that BGT treatment cause cells cycle arrest at G2/M phase and promotes the mitochondrial dependent apoptosis in A549 cells.

Since BGT exhibited better anti-cancer effect and had no safety issues, we further proceeded to identify the active principle by bio-activity guided fractionation approach. Initially, column chromatography was performed for partial purification of BGT. The 6 active fractions which were eluted were pooled as P1& P2. Most importantly, both P1 & P2 exhibited increased cytotoxicity in A549 cells, when compared with BGT. These findings are supported by earlier reports wherein cytotoxicity effect of purified *piper petal* leaf extract was higher than their crude extract^35^. Hence the increased cytotoxicity of P1 and P2 may be due to the enrichment of active principles in both P1 & P2. We further sub-fractionated the fractions with TLC and 10 subfractions (SF) were collected. Among which the SF-5 and SF-8 showed significant activity against A549 cells. LC-MS-MS analysis of SF-5 and SF-8 found two active principles in both the subfractions namely β-sitosterol (BS) and daucosterol (DS), which are categorized under the class phytosterols. BS is well documented for their anticancer property against prostate, breast and colon cancers^36^. Interestingly, recent reports suggested that anticancer mechanism of BS is similar to the activity of colchicines in binding preferentially on β-tubulin isotypes such as βII and βIII in the αβ-tubulin dimer^37^. Both the SEM and phase contrast analysis revealed that large number of mitotic cells was observed during BGT treatment. These results substantiated that the active constituents (probably BS) in BGT inhibits the mitosis of A549 cells. Similarly, BS is known for cholesterol lowering effect through prevention of cholesterol absorption in intestines. Sub-acute toxicity profile showed reduction in the level cholesterol in animals treated with the maximum concentration of BGT, which might be attributed to the presence of BS in BGT. Moreover, BS promotes the apoptosis in leukemic cancer cells U937 and HL60 cells through abrogating Bcl-2 and PI3K/Akt signaling pathways^38^. Decreased expression of Bcl-2 was also found in BGT treated A549 cells. The present study discloses the property of BGT to promote G2M phase arrest in A549 cells. Hence our future work is targeted towards understanding the role of Bcl-2 and PI3K/Akt signaling mechanism of BS against A549 cells. Indeed, vast number of anticancer effect of BS has been found in the scientific literature. Nevertheless there are no available reports on the anticancer effect of BS on NSCLC cell. Earlier study has found that BS has lesser cytotoxic effect in A549 cells when comparable with breast cancer MDA-MB-231 cells^39^.

On the other hand, the other identified compound daucosterol (DS) which is structurally similar to BS except for the addition of glycosides groups, act differently in A549 cells. It has higher cytotoxic effect than BS which shows that the apoptotic effect of BGT may be due to combination of BS and DS or DS alone. Recent study demonstrated that DS promotes autophagy in breast and gastric cancer cells by ROS dependent manner^40^. In the current study, we observed a strong growth inhibition when the cells were treated with a combination of both BS and DS. Hence when used in combination, BS and DS will be potent anticancer agents which are safe and devoid of toxicity. Taken together, the finding of the present study indicated that the benzene extract of *Grewia tiliaefolia* leaf exhibits promising anticancer and apoptotic effect on human NSCLC A549 cells. Also, the absence of toxicity in normal lung cells and animal system warrants these compounds as promising candidates in the development of potential anticancer agent for lung cancer treatment. Further investigation is being carried out to delineate the anticancer mechanism of BS and DS against A549 cells by employing both *in vitro* and *in vivo* experimental models. In addition, the effect of combination activity of BS and DS will also be assessed with special focus on microtubule organization, cell cycle arrest and apoptosis.

## Methods

### Plant collection and preparation of extracts

*G.tiliaefolia* (GT) leaves were collected from the Eastern Ghats region of India (Sirumalai hills, Dindigul, Tamil Nadu, 10.198577, 78.01353) and identified by Dr. S. John Britto, Director, The Rapinat Herbarium, Centre for Molecular Systematics, St. Joseph’s College, Tiruchirappalli, India. The assigned voucher Number is DSM001. The leaves were washed, dried, powdered and subjected to successive extraction ranging from increased polarity such as Petroleum ether, Benzene, Dichloromethane, Chloroform, Ethyl acetate, Acetone, Methanol and Water (SRL, India). Non-polar solvent extracts were dissolved in 0.01% DMSO (Sigma, USA) and DMSO was kept as internal control for cytotoxic experiments.

### Cell lines and culture

Human lung cancer cell line A549, MCF-7, PC3 and L-132 were obtained from National Centre for Cell Science (NCCS, Pune, India). A549, MCF-7, PC3 and L-132 were maintained in Ham’s F12 and DMEM medium (Invitrogen, USA) respectively. The media was supplemented with 10% FBS and 1X antibiotics (Invitrogen, USA) in a humidified atmosphere with 5% CO_2_.

### Cytotoxicity assay

The cytotoxic effect of *G.tiliaefolia* leaf on A549, cells was determined by MTT (3-(4, 5- dimethylthiazol-2-yl)-2,5-diphenyltetrazolium bromide) and LDH (Lactose Dehydrogenase) assay^41^. In addition, cytotoxic effect GT extract also studied in other cancer cell lines such as MCF-7 and PC-3. After 48 h, the extract containing medium was removed and incubated with freshly prepared MTT solution [1 mg/ml] (Himedia, India) at 37 °C for 3 h. After incubation, MTT solution was removed and formazan crystal was solubilized with 0.1 ml of DMSO and the absorbance was measured at 570 nm using Multi Label Reader spec (Molecular Device Spectramax M3, equipped with Softmax Pro V5 5.4.1 software). Meanwhile, the images were taken in all the control and treated cells using phase contrast microscopy (Nikon ECLIPSE, Ti-E, Japan). Cell membrane integrity and cell viability was assessed by LDH (Himedia, India) assay. The absorbance was measured at 340 nm and the percentage of LDH leakage was calculated by comparing the absorbance with total LDH activity of untreated A549 cells which were lysed with 0.2% Triton X-100 (Himedia, India).

### PBMC isolation and cytotoxicity assessment

#### Ethical statement

Informed consent letter was obtained from all the blood donors. The experimental protocol was approved by the Institutional Ethics Committee of Alagappa University, Karaikudi, India (Approval No. IEC/AU/2014/7) and the experiments were carried out in accordance with the relevant guidelines.

Cytotoxicity of BGT in human primary PBMC cells was assessed by trypan blue dye exclusion method as described earlier^42^. The percentage of cell viability was calculated by using the formula

1 $$ \% \,{\rm{of}}\,{\rm{Cell}}\,{\rm{cytotoxicity}}=\frac{{\rm{Number}}\,{\rm{of}}\,{\rm{Live}}\,\mathrm{cells}\,}{{\rm{Total}}\,{\rm{number}}\,{\rm{of}}\,{\rm{cells}}}\,\times \,100$$

### Mutagenicity and Genotoxicity test (Ames and Comet assay)

Mutagenicity and genotoxicity of BGT was assessed by Ames and Comet assay respectively as described earlier^42^. Ames test was performed using histidine auxotrophs of *Salmonella typhimurium* strains TA98 and TA100 (MTCC, Chandigarh, India). Two positive mutagens were used, sodium azide for TA100 (1 μg/plate) and 4-nitroquinoline (0.1 μg/plate) for TA98. After incubating the plates for 3 d at 37 °C, the individual colonies were counted and mutagenicity ratio (MR) was calculated using the formula

2 $${\rm{Ratio}}\,{\rm{of}}\,{\rm{Mutagenicity}}=\frac{{\rm{Number}}\,{\rm{of}}\,{\rm{revertant}}\,{\rm{in}}\,{\rm{treated}}\,{\rm{group}}}{{\rm{Number}}\,{\rm{of}}\,{\rm{spontaneous}}\,{\rm{revertant}}\,{\rm{in}}\,{\rm{control}}}$$

Single cell gel electrophoresis/Comet assay was performed to test the DNA damaging effect of BGT in human PBMC cells described earlier^42^. The images were taken in confocal laser scanning microscopy (Model: LSM 710, Carl Zeiss, Germany). The comet score was measured using Open Comet Score (Open Comet v1.3.1) and the comet parameters like tail moment, olive moment and percentage of DNA in tail were calculated.

### *In vivo* acute and sub-acute toxicity test in mice

#### Ethical statement

The experimental protocol was followed according to Organization of Economic Co-operation and Development (OECD) guidelines 423 and 407 for acute and sub-acute toxicity methods respectively. The studies were performed after the approval of Institutional Ethics Committee of Bharathidasan University, Tiruchirappalli, India (Approval No. BDU/IAEC/2014/NE/36/18.03.2014).

Safety and toxicity of BGT was further assessed in Swiss albino mice by acute and sub-acute toxicity methods^8^. Animals (Average weight of 25–30 g) were maintained under standard conditions of temperature (25 ± 2 °C) with relative humidity (50–60%) and allowed for free access of feed and water ad libitum.

#### Acute toxicity testing

The animals were fasted for 4 h and the dose was calculated according to their body weight. BGT was prepared in a non-toxic vehicle (0.5% carboxymethyl cellulose (CMC)) and different concentrations of BGT (250, 500, 1000, 2000 & 5000 mg/kg body weight) were administrated orally to Swiss albino mice. After dosing, the animals were monitored individually for 4 h. The body weights of the animals were recorded and the blood was collected by retro orbital method for hematological and blood parameters analysis. Further, the four major organs including Lungs, Liver, Kidney and Heart were taken for histopathology analysis.

#### Sub-acute toxicity testing

The Swiss albino mice were divided into four groups of five animals each. Repeated doses of BGT (2000 & 5000 mg/kg body weight) was administered orally for 28 consecutive days and the toxicity profile was assessed similar to the protocol followed in acute toxicity test.

### DAPI staining and DNA fragmentation assay

Nuclear content changes in BGT treated A549 cells was assessed by 4′,6-diamidino-2-phenylindole (DAPI, Sigma) staining method as described by earlier^43^. Cells were stained with DAPI (10 μg/ml) for 10 min and visualized in a fluorescence microscope (Nikon ECLIPSE, Ti-E, Japan). In DNA fragmentation assay the cells were incubated with digestion buffer for O/N in −80 °C and DNA was isolated. Isolated DNA was treated with proteinase K and RNase b for O/N. Ladder pattern of DNA was analysed in 1.5% agarose gel electrophoresis at 35 V for 2 h. The gel picture was captured with gel documentation system (Bio-Rad, California, US)

### Assessment of mode of cell death by Annexin V-FITC/DAPI and AO/ETBR dual staining

The apoptotic effect of BGT was assessed through Annexin V-FITC/DAPI staining followed by fluorescence microscopy analysis. The experimental procedure has been followed as described by manufacture protocol (Biolegend, 640906). The images were taken in fluorescence microscopy for both Annexin V-FITC and DAPI and images were merged using imageJ software.

The mode of cell death in BGT treated cells were also assessed by AO/ETBR (Acridine orange/Ethidium bromide) dual staining method as described earlier^44^.

### Scanning Electron Microscopy analysis (SEM)

The surface morphology of A549 cells was analyzed by SEM. After the incubation period, the cells were fixed in 2.5% glutraldehyde in PBS for O/N. Further, cells were dehydrated with gradient ethanol 25–100% and analyzed in HRSEM (Quanta FEG 250, FEI, Eindhoven, Netherlands)

### Western blot analysis

Whole cell lysate was prepared using RIPA lysis buffer and the proteins were quantified using Bradford assay prior to SDS-PAGE. The proteins were (Control, BGT-100 & 200 μg/mL) separated on 12% gel and transferred into PVDF membrane. The membrane was blocked with 5% non-fat skim milk agar for O/N. After incubation, the membrane was incubated with appropriate primary antibody for 6 h (β-actin, Caspase-3, Caspase-9, Bcl-2, Bax- Santa Cruz Biotechnology, US. PARP, Cytochrome c from Cell Signaling Technology, Massachusetts). After incubation, Anti-rabbit IgG secondary antibody was added and incubated for 3 h. The proteins were visualized with developing solution. Images were taken in the gel documentation system and quantified using ImgaeJ software.

### Measurement of mitochondrial membrane potential (ΔΨm)

Mitochondrial mediated apoptotic effect of BGT on A549 cells was assessed by Rhodamine 123 (Sigma, USA) staining method. The cells were washed and stained with Rhodamine 123 (10 μg/ml, Sigma) for 10 min and analysed in fluorescence spectrophotometer and microscopy.

### Cell cycle analysis

Cell cycle distributions were assessed by propidium iodide staining followed by FACS Scan Flow Cytometer (Beckton-Dickinson CA, USA). Briefly, A549 cells were harvested after 48 h treatment with BGT and it was washed with ice-cold PBS for 3 times and the cells were fixed with 70% (v/v) ethanol for O/N. The cell suspensions were then washed with ice-cold PBS, re-suspended in 50 μl RNaseA (100 μg/ml) and 50 μl PI (50 μg/ml) which was incubated in dark at room temperature for 30 min.

### Fractionation of BGT by column chromatography

BGT was fractionated using silica column chromatography. The column (3.5 cm × 45 cm) was filled with silica gel (mesh size 60–120) and BGT–adsorbed silica mixture was added to the top of silica column. Fractions were eluted in the order hexane < benzene < ethyl acetate < methanol. Totally, 17 fractions were eluted which were further tested against A549 cells using MTT assay.

### Sub fractionation of BGT using TLC

The column fractions having cytotoxic activity (MTT assay) against A549 cells were pooled together which is labeled as pooled I (PI-with F8, F9) and pooled II (PII- with F14, F15), The PI and PII were tested against A549 cells by MTT and LDH assay to ensure their activity. Further, PI and PII was separated in precoated TLC plates (Merck, Germany) using binary mobile phase of 10% ethyl acetate in petroleum ether and 30% acetone in chloroform respectively.

### Identification of active constituents by LC-MS-MS

The active sub-fractions obtained from TLC were subjected to LC-MS-MS analysis (API 4000™ LC/MS/MS) with a gradient binary mobile phase of acetonitrile and 0.1% formic acid in Milli-Q water for 10 min. The obtained mass spectrum was matched with scientific literatures and active constituents were identified. The cytotoxicity of active compounds was assessed by MTT assay.

### Statistical analysis

All the experiments were conducted in triplicate (n = 3) and one-way ANOVA (using SPSS 17 statistical software) was used to compare the mean values of each treatment. Significant differences between the means of parameters were determined by using the Duncan test.
